# Supplementary figures and images for: Concordance between whole exome sequencing of circulating tumor DNA and tumor tissue
Source: PLoS One. 2023 Oct 25;18(10):e0292879. doi: 10.1371/journal.pone.0292879 (PMC10599540; doi:10.1371/journal.pone.0292879)

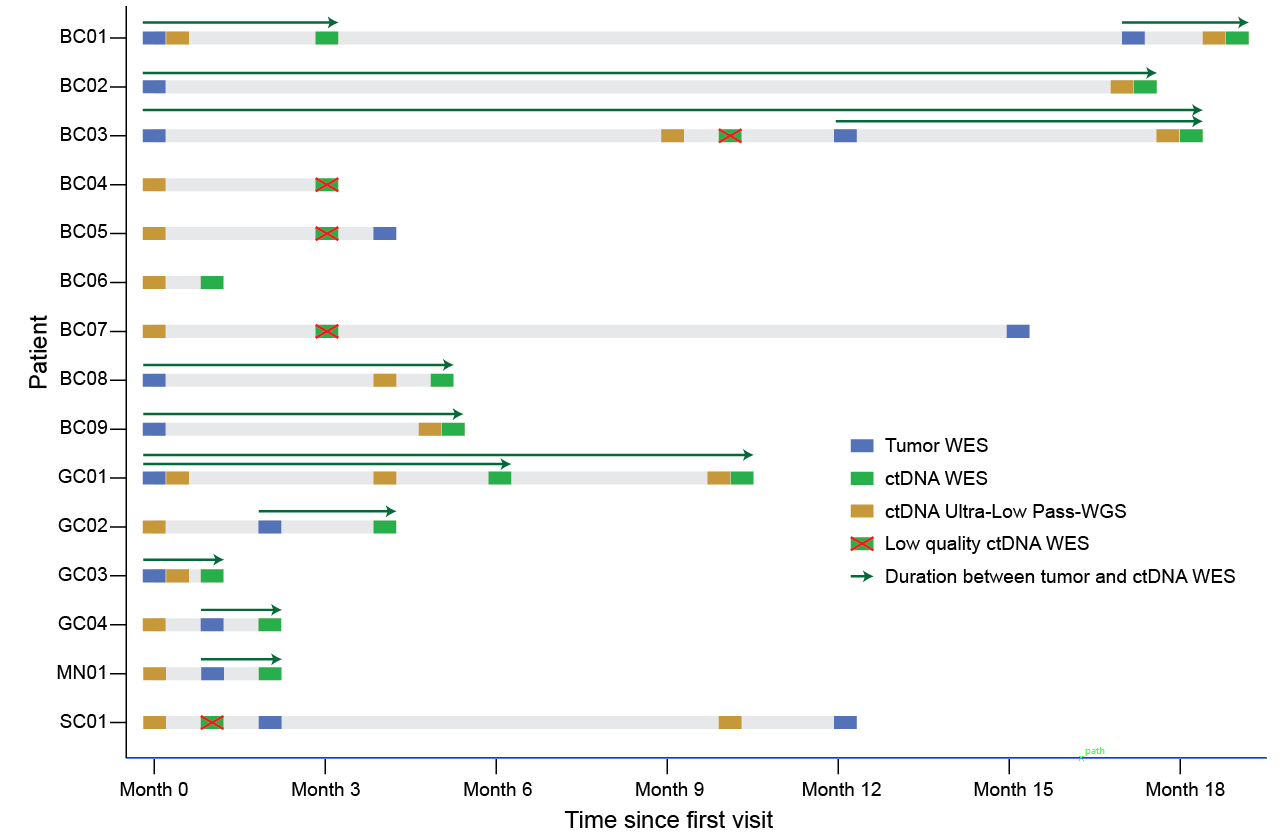

Supplement: S1 Fig — Time intervals between tumor tissue WES and ctDNA WES are indicated. Low quality ctDNA WES from samples with low ctDNA fractions were not considered in subsequent analyses. (TIF) [file pone.0292879.s001.tif]

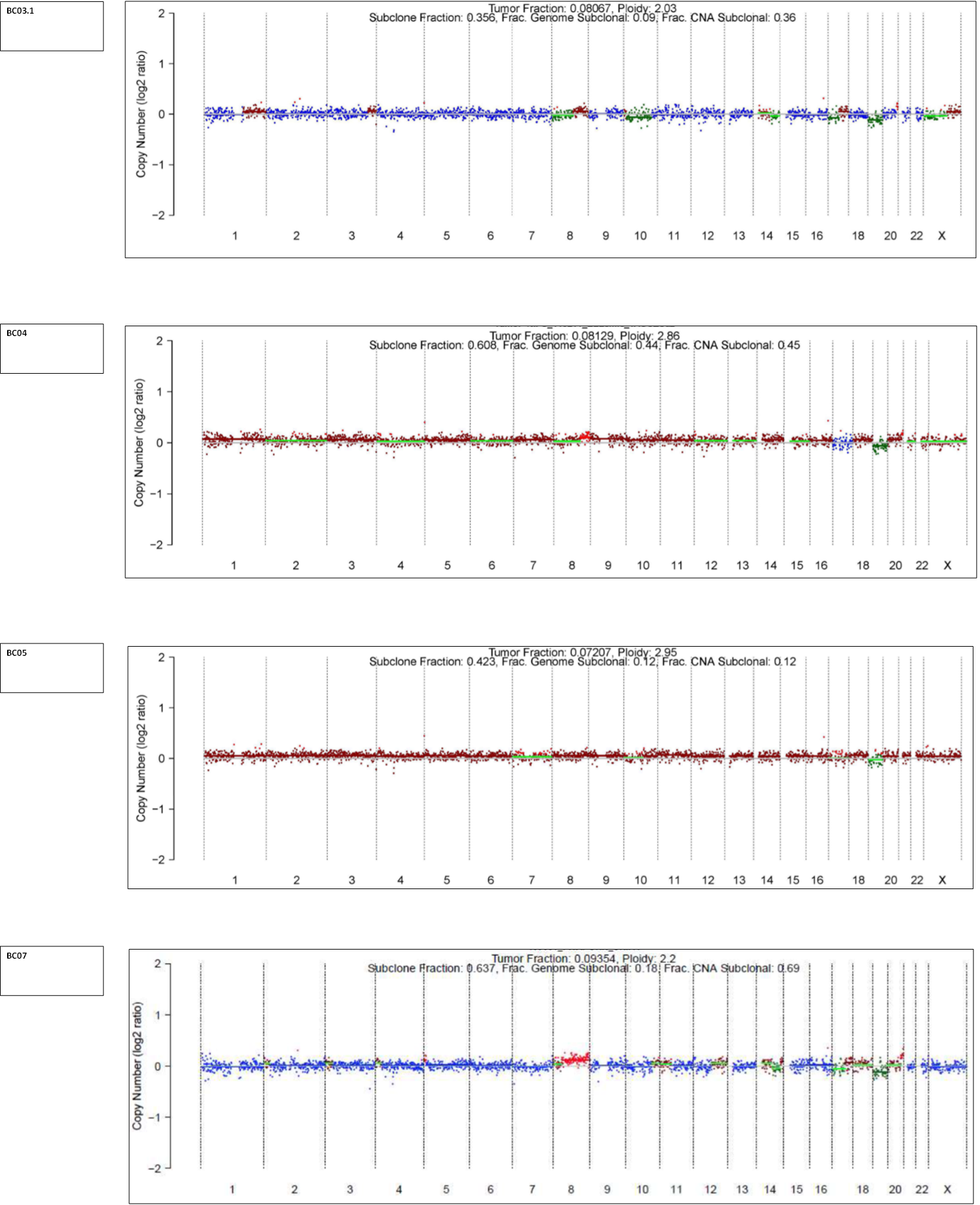

Supplement: S2 Fig — These patients were excluded from subsequent concordance analyses. (TIF) [file pone.0292879.s002.tif]

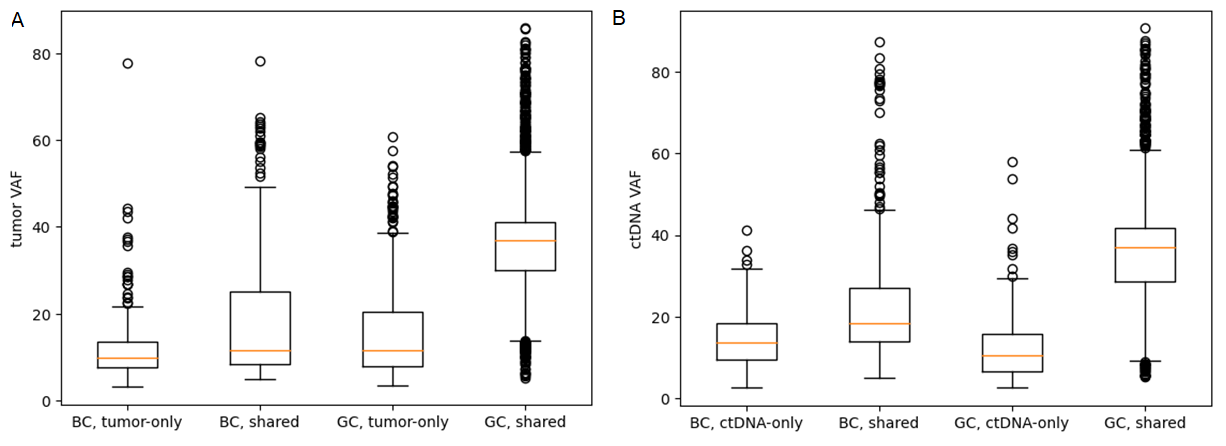

Supplement: S3 Fig — A) VAFs calculated from WES of tumor tissue. B) VAFs calculated from WES of ctDNA. Data from breast cancer (BC) and gastrointestinal cancer (GC) were shown separately as the two cancer types exhibit different VAF levels. All comparisons of VAFs between shared (concordant) and tumor-only or ctDNA-only (discordant) are statistically significant (Mann-Whitney U test p-values < 2.14e-5). (TIF) [file pone.0292879.s003.tif]

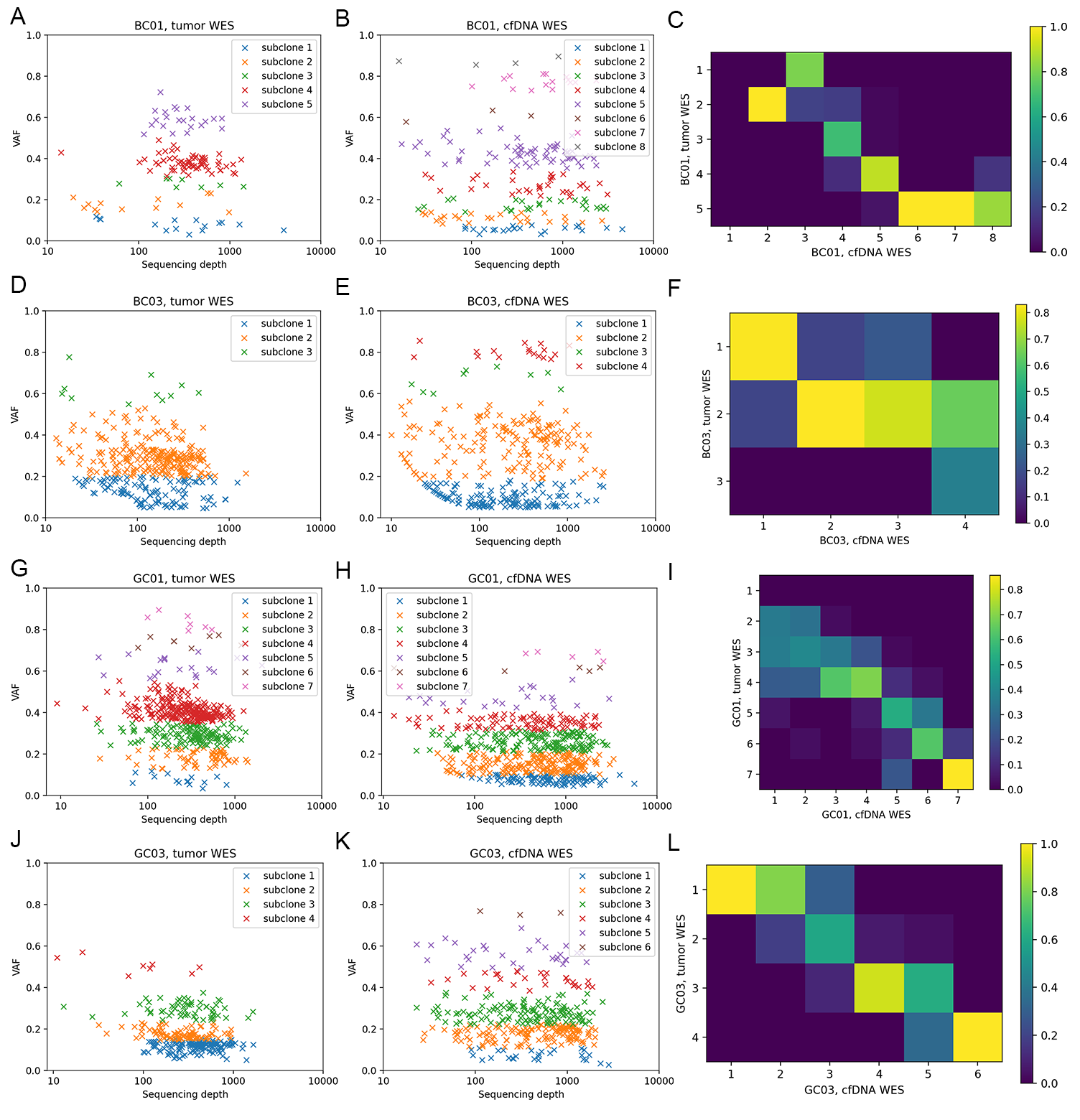

Supplement: S4 Fig — A) Scatter plot showing sequencing depths and VAFs for variants identified in BC01 patient’s tumor sample. Coloring indicates different subclones. B) Scatter plot showing sequencing depths and VAFs for variants identified in BC01 patient’s ctDNA sample. C) Heatmap showing the relative Jaccard Index between subclones identified in tumor and ctDNA samples from BC01 patient. D-F) Similar plots for BC03 patient. G-I) Similar plots for GC01 patient. J-L) Similar plots for GC03 patient. (TIF) [file pone.0292879.s004.tif]
