# Supplementary material for: Concordance between whole exome sequencing of circulating tumor DNA and tumor tissue
Source: PLoS One. 2023 Oct 25;18(10):e0292879. doi: 10.1371/journal.pone.0292879 (PMC10599540; doi:10.1371/journal.pone.0292879)
Supplement: Attachment — Submitted filename: Review-of-PONE-D-23-16936.docx [file pone.0292879.s006.docx]

Review of PONE-D-23-16936: *Concordance between whole exome sequencings of circulating tumor DNA and tumor tissue* by Leenanitikul, J et al.

Review of PONE-D-23-16936: Concordance between whole exome sequencings of circulating tumor DNA and tumor tissue by Leenanitikul, J et al.

In this manuscript the authors have conducted a validation study of whole-exome sequencing (WES) on paired ctDNA and tumor biopsy in 15 cancer patients to assess the extent of concordance between mutational profiles derived from the two source materials. The primary conclusion of the study is to counter previously a published report that that have stated a much lower cutoff for ctDNA fraction at 3% and recommend instead a range of 16-30% cutoff.

Figure 1 and Table 1 are clear and interpretable. Figure 2 needs to be larger and have legends for the different color-coded points in the scatter-graph. On line 206 the authors state that “There is a clear positive correlation between ctDNA fraction and the number of identified variants (Figure 2A, Pearson’s correlation = 0.4657, p-value = 0.069).” However, the correlation coefficient is low, and the p-value does not reach significance, this must be commented upon.

While the references list citations 34-36, these are not referred to in the text of the manuscript. Also, the Discussion should include a comparison of the authors findings in the context of other similar studies that have compared WES in ctDNA vs. tumor biopsy, such as references 34-36, which though listed in the references, are not discussed, or cited in the main text. The authors may also consider referring to Imperial et al, 2019 (PMID: 31546879), Sabatier et al 2022 (PMID: 35965534) and Manier et al 2018 (PMID: 29703982). The cut-off ranges for ctDNA fractions across these publications would also serve to bolster the authors conclusions.

Figure 4 is well presented however, again, the legends of the figure are impossibly small which makes ease of interpretation difficult. The authors should increase the size. Plus, simple titles such as “Breast cancer, n=6”; “Gastric cancer, n=3” and “Melanoma n=1” would go a long way for ease of interpretation.

The authors should discuss the clonality or sub-clonality of the mutations observed and whether it is possible capture sub-clonal variants in both methodologies. Lastly, the authors may consider discussing the clinical applicability of their findings, the clinical scenarios in which ctWES should be considered over and above that of tumor biopsy WES in the context of their findings.
